# A mutation in *CTSK *gene in an autosomal recessive pycnodysostosis family of Pakistani origin

**DOI:** 10.1186/1471-2350-10-76

**Published:** 2009-08-12

**Authors:** Muhammad Naeem, Sabeen Sheikh, Wasim Ahmad

**Affiliations:** 1 Department of Biotechnology, Faculty of Biological Sciences, Quaid-i-Azam University Islamabad-45320, Pakistan; 2 Department of Biochemistry, Faculty of Biological Sciences, Quaid-i-Azam University Islamabad-45320, Pakistan

## Abstract

**Background:**

Pycnodysostosis is a rare autosomal recessive skeletal dysplasia characterized by short stature, osteosclerosis, acro-osteolysis, frequent fractures and skull deformities. Mutations in the gene encoding cathepsin K (*CTSK*), a lysosomal cysteine protease, have been found to be responsible for this disease.

**Objectives:**

To identify pathogenic mutation in a consanguineous Pakistani family with 3 affected individuals demonstrating autosomal recessive pycnodysostosis.

**Methods:**

Genotyping of 10 members of the family, including three affected and seven unaffected individuals was carried out by using polymorphic markers D1S442, D1S498, and D1S305, which are closely linked to the *CTSK *gene on chromosome 1q21. To screen for mutations in the *CTSK *gene, all of its exons and splice junctions were PCR amplified from genomic DNA and sequenced directly in an ABI Prism 310 automated sequencer.

**Results:**

Genotyping results showed linkage of the pycnodysostosis Pakistani family to the *CTSK *locus. Sequence analysis of the *CTSK *gene revealed homozygosity for a missense mutation (A277V) in the affected individuals.

**Conclusion:**

We describe a missense mutation in the *CTSK *gene in a Pakistani family affected with autosomal recessive pycnodysostosis. Our study strengthens the role of this particular mutation in the pathogenesis of pycnodysostosis and suggests its prevalence in Pakistani patients.

## Background

Pycnodysostosis is an uncommon autosomal recessive skeletal dysplasia with a uniform clinical phenotype characterized by short stature, osteosclerosis, acro-osteolysis of the distal phalanges, frequent fractures, clavicular dysplasia and skull deformities with delayed suture closure. Less than 200 patients have been reported worldwide since the first description of the phenotype in 1962 [1]. The responsible gene was discovered by positional cloning strategy as cathepsin K (*CTSK*) on chromosome 1q21 [2]. To date, 27 different mutations, spread throughout the gene, have been reported in 34 unrelated pycnodysostosis families [3,4]. *CTSK *gene encodes a polypeptide of 329 amino acids, a member of papain-cysteine protease family and is highly expressed exclusively in osteoclasts [5]. *CTSK *is critical for bone remodeling and resorption by osteoclasts and therefore, it represents a potential target in treatment of diseases involving excessive bone loss such as osteoporosis. Cathepsin K-deficient mice generated by targeted inactivation of the *CTSK *gene display an osteopetrotic phenotype, and their ultrastructural, histological, and radiological abnormalities closely resemble those described for pycnodysostosis [6]. In the present study, we report the identification of a missense mutation (A277V) in a family of Pakistani origin with pycnodysostosis.

## Methods

### Subjects

**Figure 1:**
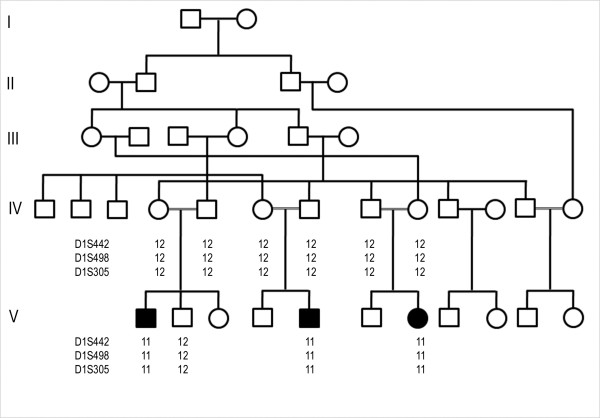
**Pedigree of the individuals affected with Pycnodysostosis**. Filled symbols identify affected subjects. Consanguineous marriages are represented with double lines. Haplotypes for the most closely linked markers are shown below each symbol.

We ascertained a Pakistani consanguineous family (Fig. 1) including three individuals affected with pycnodysostosis. The study was approved by the Institutional Review Board of Quaid-i-Azam University Islamabad, Pakistan. Informed consent was obtained from all family members who participated in the study. Family pedigree provided convincing evidence for autosomal recessive mode of inheritance and consanguineous loops accounted for all the affected persons being homozygous for the mutant allele.

### Extraction of genomic DNA and genotyping

Venous blood samples were obtained from 10 family members, including the three affected individuals. Genomic DNA was extracted from whole blood following a standard protocol, quantified by spectrophotometric measurement of optical density at 260 nm and diluted to 40 ng/μL for amplification by polymerase chain reaction (PCR). PCR amplification of microsatellite markers (D1S442, D1S498, and D1S305) was carried out according to a standard procedure in a total volume of 25 μl, containing: 40 ng genomic DNA, 20 pmol of each primer, 200 μM of each dNTP, 1 U of Taq DNA polymerase (MBI Fermentas) and 1× PCR buffer. PCR was carried out for 35 cycles, with the following thermal cycling conditions: 95°C for one minute, 57°C for one minute, 72°C for one minute, followed by final extension at 72°C for seven minutes in a thermal cycler 9600 (Perkin Elmer, Norwalk, Connecticut, USA). PCR products were resolved on 8% non-denaturing polyacrylamide gel, along with the appropriate allelic ladder, and genotypes were assigned by visual inspection.

### Mutation analysis

PCR products of coding exons (2–8) and exon/intron splice junctions of the *CTSK *gene were generated from genomic DNA, purified with Rapid PCR Purification system (Marligen Bio-sciences, Ijamsville, MD, USA) and were sequenced in an ABI Prism 310 automated sequencer, using the Big Dye Terminator Cycle Sequencing Kit (PE Applied Biosystems) following purification in a Centri-Sep Spin Column (PE Applied Biosystems). The primer sequences used are available on request. Sequence variants were identified using Bioedit, sequence alignment editor version 6.0.7.

The identified mutation obliterated an *AciI *site, so its presence was assayed by PCR amplification of cathepsin K exon 7 from genomic DNA, digestion of the product with *AciI*, and separation of the resulting fragments by agarose gel electrophoresis with direct visualization using ethidium bromide.

## Results

Clinical information was obtained for all family members with the disease phenotype. All patients presented with increased radiodensity of bones and skeletal dysplasia. They had short stature, skull deformities with frontal bossing, open sutures and fontanelles, dental malformations, hypoplasia of the mandible with obtuse mandibular angle and stubby hands and feet. The patients were mentally normal and had no psychiatric illness. The age of patient V-1 was 7 years at the time of study, while patients V-5 and V-7 both were 11 years of age. Analysis of laboratory parameters showed normal values for Hb, leukocyte number, thrombocyte number, MCV, plasma phosphate, calcium and alkaline phosphatase. Heterozygous parents were clinically indistinguishable from genotypically normal individuals.

### Genotyping and mutation analysis

**Figure 2:**
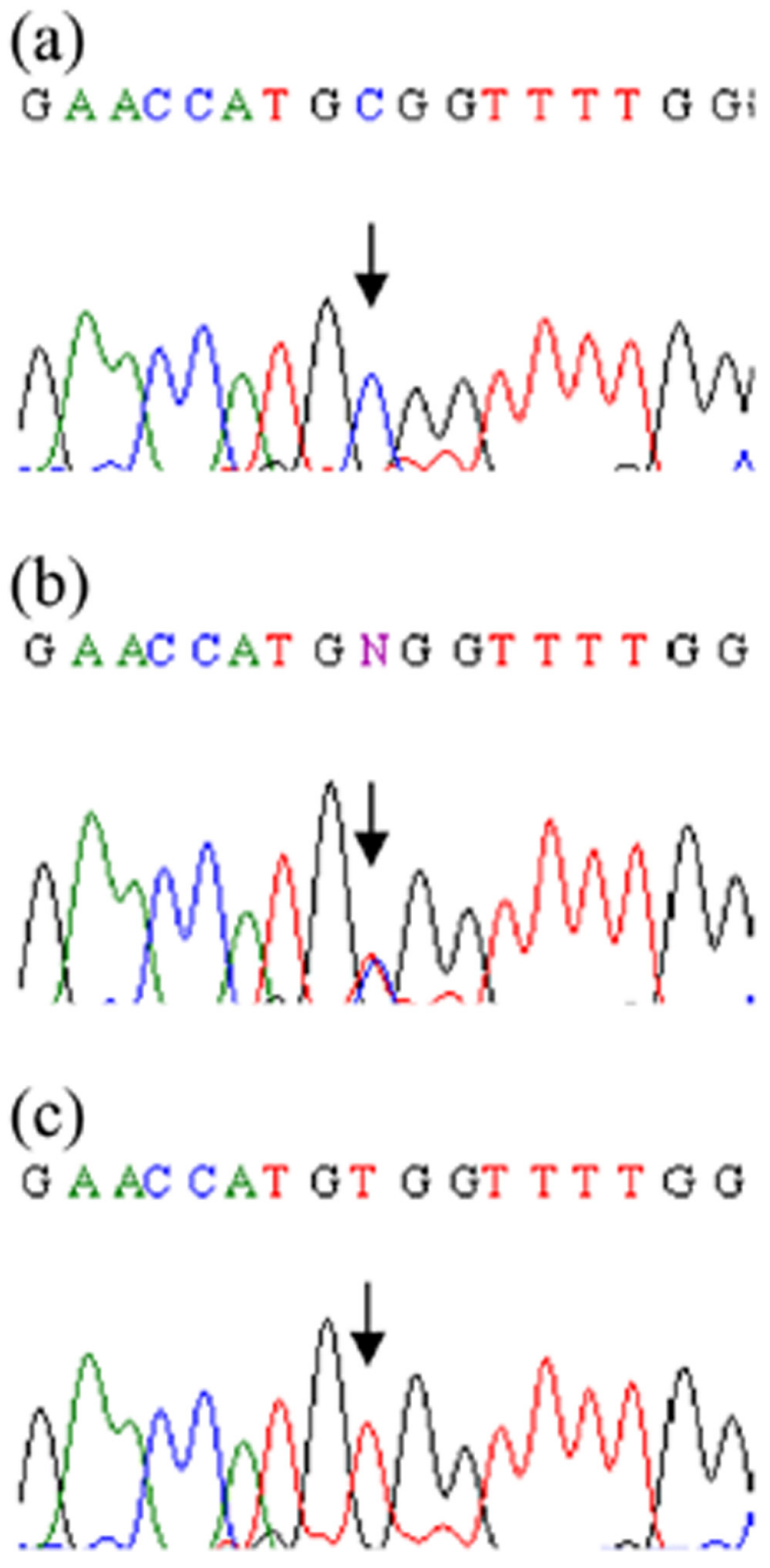
**Mutation analysis of the *CTSK *gene**. DNA sequences of exon 7 of the *CTSK *gene from (a) a control individual, (b) a heterozygous carrier and (c) a homozygous (affected) individual are shown. The arrow denotes nucleotide position 935, which is mutated from C to T, resulting in A277V missense mutation.

**Figure 3:**
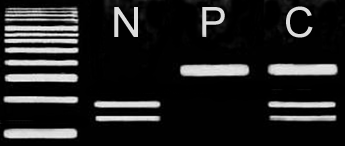
**An ethidium bromide-stained agarose gel containing 331-bp amplified product that was digested with *AciI***. The digested PCR products from a normal control individual (N), an affected individual (P) and a heterozygous carrier (C) are shown. The normal PCR product was digested into 188-bp and 143-bp fragments, whereas the 331-bp products with the A277V defect were not digested. A 100-bp DNA ladder is shown, left.

Genotyping of three affected and seven normal individuals from the family was performed with three polymorphic markers (D1S442, D1S498, and D1S305), closely linked to *CTSK *locus on chromosome 1q21. The markers were fully informative, and all the affected individuals of the family were homozygous for the markers (Fig. 1), suggesting linkage to the *CTSK *locus. The coding portion and intron-exon borders of the *CTSK *gene were then sequenced. Sequence analysis in affected individuals (V-1, V-5, V-7) revealed a homozygous C to T transition of nucleotide 935 in exon 7 (Fig. 2c), predicting an alanine to valine (A277V) amino acid substitution. This mutation was present in the heterozygous state in obligate carriers within the family (Fig. 2b). To ensure that the mutation does not represent a neutral polymorphism in the Pakistani population, 50 normal individuals were screened for mutation by direct sequencing and *AciI *restriction assay (Fig. 3). The mutation was not identified outside the family.

## Discussion

Pycnodysostosis is a potentially life-threatening genetic disease due to respiratory problems in early infancy. Maroteaux and Lamy speculated that the French artist and aristocrat Henri de Toulouse-Lautrec suffered from pycnodysostosis [1]. The gene encoding this phenotype has been mapped to human chromosome 1q21 by genetic linkage analysis, and the responsible gene was identified by a positional cloning strategy, as cathepsin K, a lysosomal cysteine protease [2].

This study presents identification of a missense mutation (A277V) in the mature Cathepsin K polypeptide in a consanguineous Pakistani family. The codon for alanine 277 in *CTSK *gene is GCG. The C to T transition at nucleotide position 935 leading to A277V amino acid substitution might be a result of methylated CpG deamination mutation of 5-methyl cytosine. Methylated CpG sequences are known to have a higher mutation rate than other dinucleotides [7]. In addition, there are two reports of an Ala to Gln substitution by C to A transition at the same site [8], suggesting that nt 935 is a mutational hotspot for pycnodysostosis. The A277V mutation has been previously reported in 5 unrelated patients, two of whom are of Pakistani origin [3,8-10]. Identification of A277V mutation in third Pakistani family suggests its prevalence in this population.

**Figure 4:**
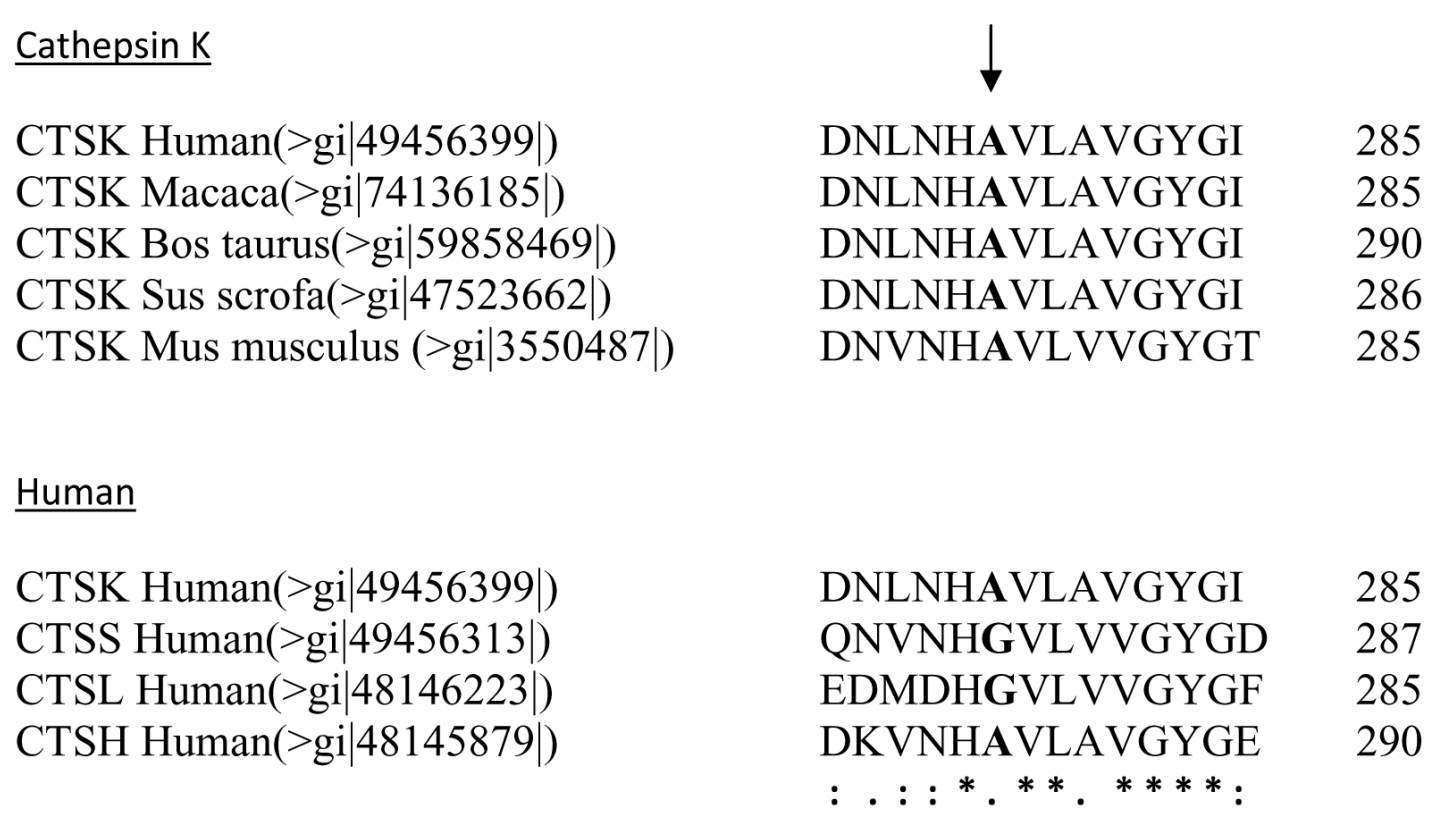
**Alignment of conserved motif from the mature region of several members of the papain family of cysteine proteases and various species**. The fourteen-residue motif from four human cysteine cathepsins (cathepsin K, cathepsin L, cathepsin S and cathepsin H) and cathepsin K of various species are aligned. The site of the cathepsin K A277V mutation is indicated by the arrow. * indicates highly conserved amino acids while. and: indicate polar → polar and polar → non polar amino acid change, respectively.

The A277 is highly conserved among papain family members, existing as either an alanine or a glycine residue (Fig. 4). The 3D structure of cathepsin K have shown that A277 residue is located in the active site immediately next to the invariably conserved H276 residue that forms the ion pair with the active cysteine (C139) to affect protein catalysis [11]. The expression studies [8] of A277V mutation resulted in a precursor protein that was unable to autoactivate and was unstable in the presence of pepsin. The obliterative effects of A277V thus suggest that there is little tolerance for larger amino acid side groups at this critical position in the active cleft.

## Conclusion

Identification of A277V mutation in Pakistani family strengthens the role of this particular mutation in the pathogenesis of pycnodysostosis and suggests its prevalence in Pakistani patients.

## Competing interests

The authors declare that they have no competing interests.

## Authors' contributions

MN studied the family, designed research plan, performed genotyping & DNA sequencing and prepared manuscript. SS performed DNA sequencing & sequence alignment. WA collected funds for study and analyzed the data. All authors read and approved the final version of the document.

## Pre-publication history

The pre-publication history for this paper can be accessed here:


